# Radio frequency blanching facilitates enhanced release of bioactive phytochemicals and boosts antioxidant capacity in apple juice from Comminuted solid-liquid mixtures

**DOI:** 10.1016/j.fochx.2026.103931

**Published:** 2026-04-27

**Authors:** Yidian Li, Shikang Tang, Wenjie Ding, Bimal Chitrakar, Baoguo Xu, Junjie Yi, Xuejiao Wang, Chaofan Guo

**Affiliations:** a Faculty of Food Science and Engineering, Kunming University of Science and Technology, Kunming 650500, China; b Yunnan Key Laboratory of Plateau Food Advanced Manufacturing, Kunming 650500, China; c Yunnan International Joint Laboratory of Green Food Processing,Kunming 650500, China; d International Green Food Processing Research and Development Center of Kunming City, Kunming 650500, China; e College of Food Science and Technology, Hebei Agricultural University, Baoding 071000, China; f School of Food and Biological Engineering, Jiangsu University, 212013 Zhenjiang, Jiangsu, China

**Keywords:** Radio frequency blanching, Bioactive compound migration, Quality preservation, Flavor retention, Thermal processing efficiency, Resource utilization

## Abstract

Apple juice extraction generates substantial amounts of pomace rich in phenolic compounds, many of which are poorly recovered due to polysaccharide-phenolic interactions and limited mass transfer. Here, radio frequency (RF) blanching was applied to comminuted apple solid-liquid mixtures to enhance bioactive phytochemical release and antioxidant capacity in pressed juice, compared with water-bath (WB) blanching. Under the RF-9 treatment condition (9 cm electrode gap), the mixture heated from 27 to 70 °C in 8 min (WB: 25 min). RF-9 increased total phenolics and flavonoids to 29.35 mg GAE/100 mL (+90.5%) and 21.75 mg RE/100 mL (+222.2%), with ∼50% higher DPPH/ABTS activity. Browning was suppressed (L* 47.35, +37%). GC–MS showed better retention of fruity esters/alcohols and reduced aldehyde off-notes, supported by SEM-observed cell wall disruption. Overall, RF blanching shows potential as a rapid pretreatment to improve pressed apple juice quality and enhance the transfer of bioactive compounds into the juice phase.

## Highlights

• RF blanching speeds heating of apple solid–liquid mix.
• RF blanching inhibits PPO/POD and reduces browning in pressed juice.
• RF blanching boosts phenolic release and antioxidant capacity in juice.
• RF blanching better preserves volatiles and improves sensory quality.

## 1 Introduction

Apple is one of the world's most important economic crops, and the industrial juicing process generates a large amount of pomace as a byproduct. This pomace, which consists of fruit pulp, peel, and seeds, still contains valuable bioactive compounds, but these are often underutilized in the current production process (Bhushan et al., 2008). The industrial apple juice production line typically involves sorting, comminution, blanching, pressing, and subsequent clarification and filtration. During comminution, the fruit is reduced to 3–6 mm particles, creating a solid-liquid mixture that is then subjected to blanching. However, despite containing numerous bioactive components such as phenolic acids (e.g., chlorogenic acid derivatives), flavonoids (e.g., epicatechin and quercetin), procyanidins, and organic acids, the solid phase of this mixture, including both the flesh and peel, is not fully utilized (da Silva et al., 2020; Da Silva et al., 2023). These bioactive compounds are associated with various health benefits, including antioxidant, anti-inflammatory properties, and the prevention of non-communicable chronic diseases, among other potential effects (Bondonno et al., 2017).

Blanching is a short-term heat treatment process that uses hot water or saturated steam to treat fruit and vegetable materials. This operation rapidly inactivates endogenous enzyme systems (such as polyphenol oxidase) and some microorganisms while simultaneously achieving tissue softening and color stabilization, thus providing a quality assurance foundation for subsequent juicing and sterilization processes (Barry-Ryan, 2012; Lemmens et al., 2009). Traditional blanching technology has been primarily applied to minimally processed products, characterized by high temperatures, long heating times, and low heating efficiency. It may be associated with undesirable side effects, including vitamin degradation, off-flavors, color changes, and the loss of heat-sensitive compounds (Geysen et al., 2005). If the blanching process after comminution is improved to enhance the release efficiency of polyphenols from the solid phase, and if this treatment can effectively preserve and improve the quality of apple juice, it would subsequently increase the utilization of pomace and enhance the nutritional value of the juice. Radio frequency (RF) heating technology, as a novel thermal treatment method in food processing, has demonstrated significant potential in the blanching of fruit and vegetable products due to its deep penetration and efficient energy conversion characteristics (Hao et al., 2021). This technology is particularly suitable for the thermal processing of fruit and vegetable foods. In the case of high-viscosity apple solid-liquid mixtures, which are rich in pectin and dietary fiber, the deep penetration ability of RF has been reported to alleviate temperature gradient problems associated with conventional heat transfer in high-viscosity food systems (Huang et al., 2024). Theoretically, this approach offers a new pathway to maximize the retention of heat-sensitive nutritional components during the blanching process. This method not only preserves the heat-sensitive constituents in the fruit pulp but also maintains its sensory and nutritional quality. In our previous studies, radio-frequency (RF) heating increased the levels of bioactive compounds in the liquid phase of passion fruit pulp solid-liquid mixtures (Sun et al., 2025). A similar effect was observed during RF processing of kiwifruit puree (Lyu et al., 2018). These findings motivated the present work to examine the feasibility of applying RF blanching to comminuted apple solid-liquid mixtures prior to juice pressing, a processing context that has received limited attention.

This study investigated the effects of radio frequency (RF) blanching on comminuted apple solid-liquid mixtures prior to juice pressing, with conventional water-bath blanching used as a reference. Freshly prepared apple solid-liquid mixtures were treated by RF blanching at different electrode gaps, followed by pressing, and compared with water-bath-treated and untreated samples. Key quality indicators, including phenolic and flavonoid contents, volatile composition, enzyme activity, and sensory attributes, were evaluated. In addition, scanning electron microscopy (SEM) was used to assess structural changes in the apple pomace after treatment. This work aims to clarify how different RF blanching conditions affect pressed apple juice quality in this specific processing context, particularly with respect to the release of bioactive compounds from the solid phase into the juice.

## 2 Materials and methods

Red Fuji “Sugar Heart” apples used in this study were purchased from Zhaoyang District, Zhaotong City, Yunnan Province, China. Apples with uniform size, regular shape, similar maturity, and no visible browning or mechanical damage were selected. All apples were stored at 4 °C before use. Before treatment, the apples were washed, dried, cored, and destemmed, and then ground with a blender to obtain an apple solid-liquid mixture.

The untreated apple solid-liquid mixture was used as the control (CK). For both water bath (WB) and radio frequency (RF) treatments, 200 g of the apple solid-liquid mixture was packed in PVE plastic sealing film and placed in a disposable food container (12 cm × 7.5 cm × 5 cm, without lid). According to the sample mass and container dimensions, the resulting sample layer height was approximately 2.1–2.3 cm. The initial sample temperature was approximately 27 °C. For WB treatment, the packaged sample was heated in a water bath at 75 °C until the temperature at the center region of the sample reached 70 °C. For RF treatment, an HGJL-6RFS device (Hefei Hag Jinlong Equipment Technology Co., Ltd., Hefei, Anhui, China) was used. The RF system operated at 27.12 MHz with a maximum output power of 6 kW and an electrode size of 450 × 600 mm^2^. The sample container was placed centrally between the upper and lower electrodes, and electrode gaps of 9 cm (RF-9), 11 cm (RF-11), and 13 cm (RF-13) were applied. A direct readout of voltage or electric field strength was not available from the RF system. Therefore, electric field intensity was not directly quantified in the present study, and the effect of electrode gap was interpreted mainly from the observed heating behavior and temperature-time profiles, with reference to RF heating theory reported in previous studies (Altin et al., 2025; Tasci et al., 2024). No stirring or mixing was performed during either WB or RF treatment. Sample temperature was monitored using a fiber-optic probe inserted into and fixed at the center region of the sample, and the temperature was recorded at 15 s intervals. Heating was terminated immediately when the sample center reached 70 °C. After heating, the samples were cooled to 30 °C and then juiced for subsequent analysis. The temperature-time curves are presented as the mean of three independent measurements.

### 2.1 Color measurement

Color measurements were performed using an Agera colorimeter (Hunter Associates Laboratory, Inc., Fairfax, USA). Before each measurement, the instrument was calibrated using black and white standard ceramic plates. Approximately 20 mL of sample was transferred into a quartz cuvette with a black lid for color determination. The L*, a*, and b* values were recorded, and the total color difference (Δ*E*) was calculated using Eq. (1):

(1) $$\Delta E=\sqrt{{\left(L_{1}^{*}-L\right)}^{2}+{\left(a_{1}^{*}-a\right)}^{2}+{\left(b_{1}^{*}-b\right)}^{2}}$$

With the control group as the reference. In this equation, Δ*E* represents the total color difference between the sample and the control; L*_1_, a*_1_, and b*_1_ represent the lightness, redness, and yellowness of the sample, respectively; and *L*, *a*, and *b* represent the corresponding values for the control sample.

### 2.2 Polyphenol oxidase (PPO) and peroxidase (POD) activity

Equal volumes of apple juice and 0.2 mol/L phosphate buffer (pH 6.5) were mixed, followed by the addition of 4% PVPP, 1% Triton X-100, and 1 mol/L NaCl. The mixture was vortexed, left to stand for 10 min, and then centrifuged at 11,000 ×*g* for 20 min at 4 °C. The supernatant was collected as the crude enzyme extract for PPO and POD activity assays. For PPO activity, 150 μL of the crude enzyme extract was mixed with 1.5 mL of 0.05 mol/L phosphate buffer (pH 6.5) containing 0.07 mol/L catechol, and the absorbance at 420 nm was recorded continuously for 10 min. For POD activity, 200 μL of the crude enzyme extract, 200 μL of 1% *o*-phenylenediamine prepared in phosphate buffer, and 200 μL of 1.5% hydrogen peroxide were added in sequence to 1.5 mL of 0.05 mol/L phosphate buffer, and the absorbance at 485 nm was recorded continuously for 10 min. Phosphate buffer was used as the blank.

Enzyme activity was calculated from the slope of the initial linear region of the absorbance-time curve after blank correction and expressed as U/mL of crude enzyme extract. One unit (U) of PPO or POD activity was defined as an increase of 0.001 in absorbance per minute. Therefore, PPO and POD activities were calculated as:

(2) $$\mathrm{PPO}\;\text{activity}\;\left(U/\mathrm{mL}\right)=\frac{\Delta A_{420}/\min}{0.001\times 0.150}$$

(3) $$\mathrm{POD}\;\text{activity}\;\left(U/\mathrm{mL}\right)=\frac{\Delta A_{485}/\min}{0.001\times 0.200}$$

where Δ*A*/min is the change in absorbance per minute, and 0.150 mL and 0.200 mL are the volumes of crude enzyme extract used in the PPO and POD assays, respectively.

### 2.3 Quantitative measurement of total phenolic content (TPC), total flavonoid content (TFC), and antioxidant activity

The determination was carried out according to the method of Silva et al. (2019), with slight modifications. Apple juice was mixed with 80% methanol at a ratio of 1:4 (*v*/v) and extracted by ultrasonication for 20 min. The mixture was then centrifuged at 4 °C and 8000 rpm for 5 min, and the supernatant was collected as the methanol extract for the determination of TPC, TFC, DPPH, and ABTS. TPC and TFC were determined by the Folin-Ciocalteu method and the aluminum chloride colorimetric method, respectively. TPC was expressed as mg gallic acid equivalent (GAE) per 100 mL of sample, and TFC was expressed as mg rutin equivalent (RE) per 100 mL of sample. DPPH and ABTS radical scavenging activities were determined using Trolox as the standard and expressed as mmol Trolox equivalent (TE) per L of apple juice.

### 2.4 Measurement of pH, total soluble solids (TSS), titratable acidity (TA), and total sugar

Before measurement, all samples were thoroughly shaken to ensure uniformity. The pH of each sample was measured at room temperature using a pH meter (FE28-Standard, Mettler Toledo Co., Ltd., Shanghai, China). Total soluble solids (TSS) were determined with a digital refractometer and expressed as °Brix. Titratable acidity (TA) was measured using an automatic potentiometric titrator (907 Titrando, Metrohm, China). The sample was titrated with 0.1 mol/L NaOH to an endpoint of pH 8.1, and the volume of NaOH consumed was recorded. TA was expressed as citric acid equivalent and calculated according to Eq. (4) (Cao et al., 2019):

(4) $$\mathrm{TA}\left(\%\right)=\frac{C\times V\times K}{m}\times 100\%$$

In this equation, *V* is the volume of NaOH consumed during titration (mL), *C* is the concentration of NaOH (0.1 mol/L), *m* is the mass of the juice sample (g), and *K* is the conversion factor for citric acid (0.067). All measurements were performed in triplicate.

Total sugar content was determined by the phenol‑sulfuric acid method. The apple juice sample was diluted 50-fold with distilled water. Then, 1.0 mL of the diluted sample was transferred into a 15 mL test tube, followed by the addition of 1.0 mL of 5% phenol solution. After that, 5.0 mL of 72% sulfuric acid was rapidly added. The mixture was allowed to stand for 10 min, vortexed thoroughly, and then kept in a 30 °C water bath for 20 min. The absorbance was measured at 490 nm. A glucose standard curve was used for quantification, and the total sugar content of apple juice was expressed as g glucose equivalent per 100 mL (g GE/100 mL).

### 2.5 Measurement of organic acids content

Organic acids were determined according to the method of Li et al. (2020), With minor modifications. A mixture containing 250 μL of 15% (*w*/*v*) K₄[Fe(CN)₆] and 250 μL of 15% ZnSO₄ was prepared, and then 5 mL of apple juice sample was added. After mixing, the mixture was allowed to stand for 30 min and then centrifuged at 10,000 ×*g* for 20 min. The supernatant was collected as the extract. Before chromatographic analysis, each extract was diluted 10-fold with HPLC-grade water and filtered through a 0.45 μm membrane. Organic acids in apple juice were analyzed using a high-performance liquid chromatography (HPLC) system (Vanquish, Thermo Fisher Scientific). The injection volume was 30 μL. The mobile phase was 25 mmol/L potassium dihydrogen phosphate buffer (pH 2.5), and the flow rate was 0.8 mL/min. Detection was carried out at 210 nm using a UV-DAD detector. Each extract was analyzed in triplicate. External standards, including oxalic acid, lactic acid, malic acid, citric acid, acetic acid, and tartaric acid, were used for quantification.

### 2.6 Scanning electron microscopy (SEM)

Microstructural changes in apple pomace after different treatments were observed by scanning electron microscopy (SEM). The treated apple pomace samples were first freeze-dried, then mounted on aluminum stubs using conductive carbon tape, and sputter-coated with gold at 20 mA for 120 s. SEM observation was performed using an Apreo 2S microscope (Thermo Fisher Scientific, Waltham, MA, USA) at an accelerating voltage of 5–10 kV and a beam current of 7. Images were collected at a magnification of 2000 × .

### 2.7 Volatile compound analysis

#### 2.7.1 Electronic nose analysis

Volatile compounds in apple juice were analyzed using an electronic nose system (Shanghai Baosheng Industrial Development Co., Ltd., Shanghai, China). Approximately 15 mL of each sample was transferred into a 50 mL headspace vial. The measurement conditions were as follows: cleaning time, 120 s; detection time, 90 s; and carrier gas flow rate, 1 L/min.

#### 2.7.2 SPME and GC–MS analysis

Volatile compounds were analyzed by headspace solid-phase microextraction coupled with gas chromatography–mass spectrometry (HS-SPME-GC–MS) using an Agilent 7000E triple quadrupole system (Agilent Technologies, Santa Clara, CA, USA). Volatile compounds were extracted from apple juice using Solid-Phase Microextraction (SPME) with a 50/30 μm DVB/CAR/PDMS fiber (Supelco, Bellefonte, USA), following a modified method by (Schmutzer et al., 2014), with slight modifications. Briefly, 5 mL of apple juice was transferred into a 20 mL headspace vial, and 20 μL of cyclohexanone was added as the internal standard. The sample was equilibrated at 55 °C for 5 min and then stirred for 25 min for volatile adsorption. After extraction, the SPME fiber was inserted into the GC injector and desorbed at 250 °C for 5 min. The fiber was then cleaned at 250 °C for 5 min after each run. n-Alkanes (C5-C25) were used to calculate the retention index (RI).

Chromatographic separation was carried out on an HP-5MS capillary column with helium as the carrier gas at a constant flow rate of 1.2 mL/min under splitless injection conditions. The oven temperature was initially set at 40 °C and held for 5 min, then increased to 120 °C at 4 °C/min, followed by heating to 250 °C at 10 °C/min and holding for 6 min. The mass spectrometer was operated in the scan mode over an *m*/*z* range of 33–500. The ion source and quadrupole temperatures were set at 230 °C and 150 °C, respectively. Qualitative identification was performed by comparing retention indices and mass spectra with those in the NIST library, and quantitative analysis was carried out using the internal standard method.

### 2.8 Sensory evaluation

Sensory evaluation was carried out as a preliminary screening by QDA with six panelists. Before the test, the panelists were introduced to the sensory attributes and the scoring criteria. Samples were served in glass cups at room temperature and identified with random three-digit codes. The serving order was randomized for each panelist, and the panelists were not informed of the treatment identity. Water was provided for mouth rinsing between samples. Each attribute was scored on a 5-point scale, where 0 meant the attribute was absent and 5 meant it was very strong. Final scores were expressed as the mean values given by the panelists. All procedures involving human participants complied with the Declaration of Helsinki, and informed consent was obtained from all participants.

### 2.9 Data processing

All measurements were performed in triplicate, and the results are presented as mean ± standard deviation. Statistical analysis was performed using SPSS 19.0. Differences among treatments were analyzed by one-way ANOVA, followed by Tukey's HSD test for multiple comparisons. Normality and homogeneity of variance were checked before analysis. Statistical significance was set at *p* < 0.05. Figures were prepared using Origin 2024.

## 3 Results and discussion

The heating rate of apple solid-liquid mixture under different thermal modes.

**Fig. 1:**
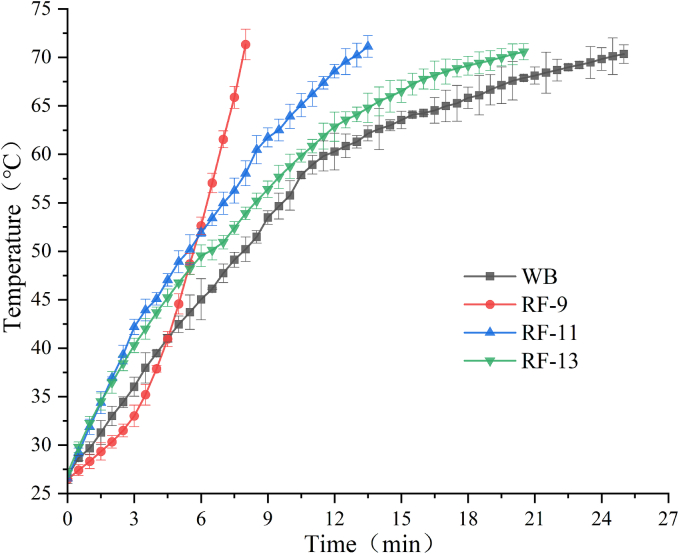
Temperature-time profiles measured at the center region of apple solid-liquid mixtures during WB and RF treatments.

As shown in Fig. 1, RF treatment markedly increased the heating rate of the apple solid-liquid mixture compared with conventional WB treatment. At an electrode gap of 9 cm (RF-9), the sample reached 70 °C in 8 min, whereas WB treatment required 25 min to reach the same target temperature. Moreover, decreasing the electrode gap from 13 cm to 9 cm further accelerated heating, with RF-13 requiring approximately 19 min to reach 70 °C. This finding suggests that a smaller electrode gap improved the heating performance of the RF system.

According to RF heating theory, when heat loss is ignored, the absorbed power of the load is related to the transient heating rate and electric field intensity, as shown in Eq. (5):

(5) $$P=\rho c_{p}\frac{\partial T}{\partial t}=2\mathrm{\pi f}\varepsilon_{0}\varepsilon^{\prime \prime }{\left|E_{m}\right|}^{2}$$

where *P* is the absorbed power of the load, *ρ* is the density, *c*_*p*_ is the heat capacity, *∂T*/*∂t* is the transient heating rate, *f* is the RF frequency, *ε*_*0*_ is the permittivity of vacuum, ε'′ is the dielectric loss factor, and *|E*_*m*_*|* is the electric field intensity in the sample. In the present study, the RF-treated apple solid-liquid mixtures had the same sample mass, formulation, container geometry, and RF frequency. Therefore, *ρ*, *c*_*p*_, *f*, and *ε*_*0*_ were considered constant, while *ε'′* was assumed to be relatively comparable within the same material system. Under these conditions, a higher heating rate indicates stronger RF heating intensity and may also reflect a relatively higher electric field intensity in the sample.

Similar trends have been reported in previous studies, where reduced electrode gaps strengthened electromagnetic coupling and enhanced dielectric heating of food materials (Costa & Marra, 2024; Tasci et al., 2024). Therefore, the faster heating observed at the smaller electrode gap in this study suggests that reducing the electrode gap enhanced the RF heating effect. However, because the RF system used in this study did not provide a direct readout of voltage or electric field strength, and dielectric properties were not directly measured, this relationship is discussed here only as a relative interpretation rather than a quantitative determination of electric field intensity. Overall, RF treatment shortened the time required to attain the target center temperature.

The juice color changes after thermal treatment of the apple solid-liquid mixture.

**Fig. 2:**
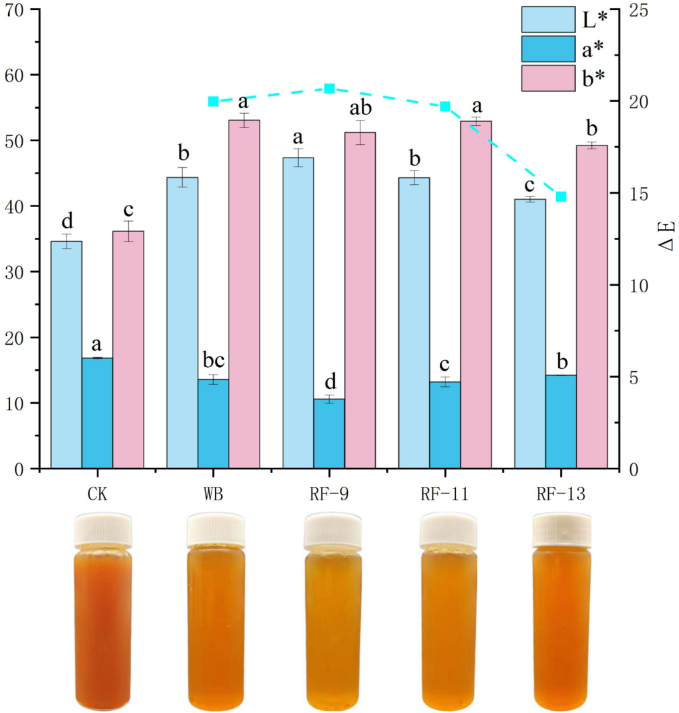
Comparative color profiles of apple juice under different treatments.

Apple juice color is an important quality attribute because it directly affects consumer acceptance and also reflects changes that occur during processing. In apple juice, phenolic compounds are easily oxidized and undergo enzymatic browning, mainly through the action of PPO and POD, leading to the formation of quinones and subsequent color deterioration. Different processing methods therefore influence juice color to different extents (Zhu et al., 2024). As shown in Fig. 2, Among all treatments, RF-9 gave the highest L* value (47.35 ± 1.37), which was 37% higher than that of the control, and the lowest a* value (10.59 ± 0.64), which was 37% lower than that of the control. This suggests that RF-9 helped maintain a lighter appearance and reduced browning. The b* value of the RF-9 group (51.19 ± 1.84) was also slightly lower than that of the WB group (53.06 ± 1.09), indicating less yellowing during processing, which may be related to the shorter heating time of RF treatment and the reduced extent of non-enzymatic browning (Krapfenbauer et al., 2006). The ΔE values relative to the control were 19.79 for WB, 20.67 for RF-9, 19.70 for RF-11, and 14.80 for RF-13. Among the treated samples, RF-9 showed the largest overall color difference from the control. Combined with its higher L* and lower a* values, this result indicates that RF-9 produced a brighter and clearer juice appearance. By contrast, RF-13 showed a smaller change in color parameters, which may be due to its weaker heating intensity and less effective inhibition of browning reactions. Taken together, RF-9 showed a higher L* value and a lower a* value than the other treated samples, indicating a lighter appearance and reduced browning under this condition, which was also consistent with the sensory evaluation results.

The PPO and POD inactivation in juice after thermal treatment of the solid-liquid mixture.

**Fig. 3:**
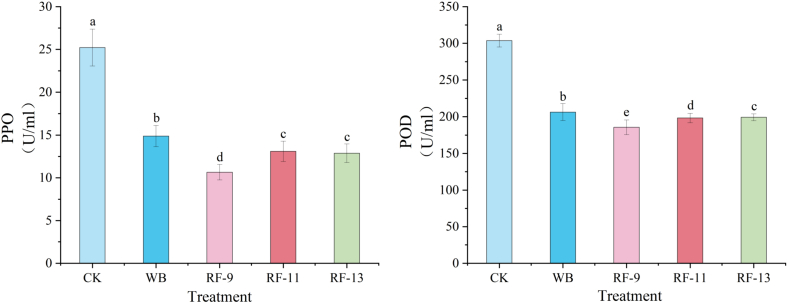
Polyphenol oxidase (PPO) (A) and peroxidase (POD) (B) activities in apple juice under different treatments.

PPO and POD are closely related to enzymatic browning in apple juice and are therefore useful indicators of color stability during processing. PPO mainly catalyzes the oxidation of endogenous phenolic compounds to quinones, which then undergo further non-enzymatic reactions to form brown pigments. POD can also participate in the oxidation of phenolic substrates in the presence of hydrogen peroxide and may further promote browning during processing (Murtaza et al., 2020). As shown in Fig. 3, RF-9 treatment significantly reduced both PPO and POD activities compared with the control group (CK) and the water-bath treatment (WB). PPO activity decreased from 25.22 U/mL in CK to 10.67 U/mL after RF-9 treatment, while POD activity decreased from 303.67 U/mL to 185.67 U/mL. In contrast, RF-11 and RF-13 showed weaker inhibitory effects, which may be related to their wider electrode gaps, lower heating rates, and weaker field intensity. The lower PPO and POD activities observed in the RF-9 group may be related to the faster heating rate of RF treatment, which reduced the time during which oxidative enzymes remained active (Manzocco et al., 2008; Zhang et al., 2020). In addition, the electric field associated with RF treatment may also contribute to enzyme inactivation to some extent, although this effect is likely secondary to the thermal effect under the present processing conditions (Manzocco et al., 2008).

Thermal treatment enhances phenolic release into the juice and increases antioxidant capacity.

**Fig. 4:**
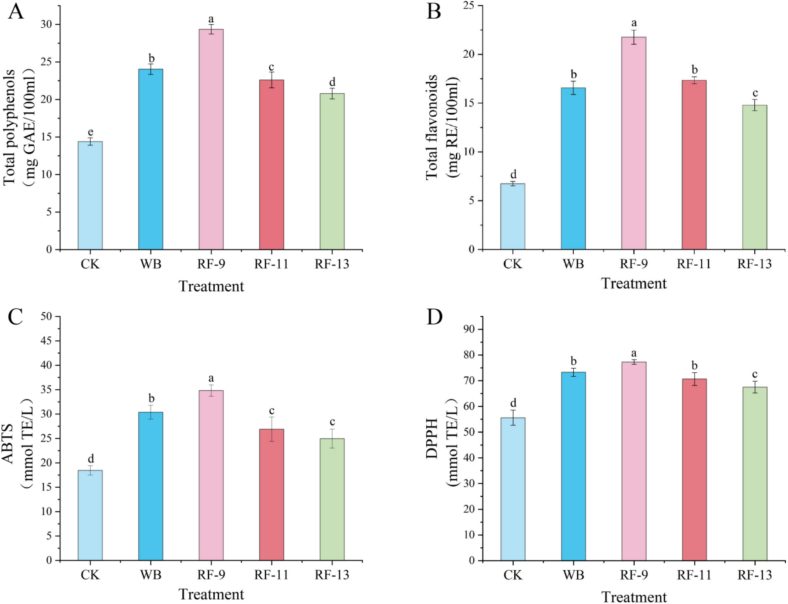
Total phenolic content (TPC) (A), total flavonoid content (TFC) (B), ABTS radical scavenging activity (C), and DPPH radical scavenging activity (D) of apple juice under different treatments.

Apples contain a large amount of polyphenols, with various phenolic compounds found in both the flesh and peel of the fruit, primarily flavonoids and phenolic acids. These substances are the main active compounds in apple juice that combat oxidative stress and cellular damage. Apple polyphenols also have potential resistance effects against cancer and inflammation (Tu et al., 2017). As shown in Fig. 4A/B, RF treatment, especially RF-9, significantly increased the content of polyphenols and flavonoids. TPC of the RF-9 sample reached 29.35 ± 0.64 mg GAE/100 mL, markedly higher than that of the control (CK: 15.40 ± 0.49 mg GAE/100 mL), and TFC also increased accordingly (RF-9: 21.75 ± 0.74 mg RE/100 mL vs. CK: 6.75 ± 0.22 mg RE/100 mL). Notably, the increase in TPC induced by RF-9 was greater than that observed with WB treatment. Previous studies have shown that prolonged heating can enhance TPC in juices such as pineapple, orange, and watermelon when treated at temperatures between 50 °C and 90 °C, a trend that is similarly reflected in the apple solid-liquid mixture in this study (Tchuenchieu et al., 2018). This increase is likely due to two main mechanisms: RF's thermal effect, which degrades the pectin network, reducing binding sites and disrupting the cellulose-pectin matrix in the apple cell walls, releasing bound polyphenols such as phenolic acid esters (Liu et al., 2019). Furthermore, RF treatment may have contributed to tissue disruption and increased permeability, which could facilitate the release of intracellular compounds such as polyphenols and organic acids (Shen et al., 2020). WB treatment, with slower heat conduction, likely results in incomplete cell wall disruption, explaining the smaller increase in TPC compared to RF-9. Additionally, the inactivation of oxidative enzymes during heat treatment reduces antioxidant compound loss. RF-9's short, high-intensity heating irreversibly inactivates PPO, minimizing phenolic compound degradation due to enzymatic oxidation (Manzocco et al., 2008). The RF-9 group also exhibited the highest antioxidant activity, with WB showing the second highest. This result may be related to faster heating and greater tissue disruption under RF treatment, which could have facilitated the release of antioxidant compounds.

The antioxidant capacity of apple juice is primarily influenced by the type and concentration of phenolic compounds, with higher levels of these compounds correlating to greater antioxidant activity, as observed by Zielinski et al. (2019). As shown in Fig. 4C/D, a positive correlation was observed between phenolic content and antioxidant capacity, evaluated through ABTS and DPPH radical scavenging assays. DPPH scavenging ability increased following heat treatment, with RF-9 samples exhibiting the highest scavenging rate, which was significantly higher than that of WB samples. This enhancement in DPPH scavenging activity could be attributed to the increased presence of strong reducing groups during RF treatment (Wu et al., 2024). RF-9 treatment, with its higher intensity and rapid heating, likely facilitated the release of antioxidant compounds by disrupting the pectin network, whereas RF-11 and RF-13 treatments, with lower intensity, may not have fully disrupted the cell walls, limiting the release of polyphenols. Both WB and RF treatments significantly enhanced the ABTS antioxidant capacity compared to the control (CK), likely due to the increased polyphenol and flavonoid content. Notably, RF-9 exhibited a more substantial improvement in ABTS activity than WB, which may reflect differences in heating behavior and release efficiency under the tested conditions. RF treatment may also have promoted the migration and release of antioxidant compounds during processing. This phenomenon has also been observed in fruits such as passion fruit, strawberry, and dragon fruit (Jiang et al., 2019; Shen et al., 2020; Sun et al., 2025).

The basic physicochemical indices after thermal treatment (TSS, pH, TA, total sugar).

TSS, pH, and TA are important basic quality indicators of fruit and vegetable products because they are closely related to sweetness, acidity, and overall sensory quality. Total sugar content is also an important index reflecting the sweetness of apple juice. As shown in Table 1, the pH values of all treated samples changed within a relatively narrow range compared with the control (CK), although RF-treated samples, especially RF-9, showed slightly lower pH values. This suggests that thermal treatment affected juice pH only to a limited extent. A similar trend was reported by (Wu et al., 2021), who found that heat treatment did not markedly alter the pH of pineapple juice.

**Table 1:** Physicochemical properties of apple juice under different treatments.

	pH	TSS (°Brix)	TA (% CAE)	Total sugar (g GE/100 mL)
CK	4.45 ± 0.02^a^	10.50 ± 0.00^c^	4.44 ± 0.16^c^	42.39 ± 0.67^d^
WB	4.41 ± 0.02^b^	11.43 ± 0.06^a^	4.58 ± 0.06^b^	53.12 ± 1.42^b^
RF-9	4.28 ± 0.02^d^	11.40 ± 0.00^a^	4.99 ± 0.32^a^	62.72 ± 3.25^a^
RF-11	4.34 ± 0.03^c^	11.23 ± 0.06^b^	4.75 ± 0.23^a^	49.52 ± 1.55^c^
RF-13	4.42 ± 0.02^b^	11.20 ± 0.00^b^	4.61 ± 0.39^b^	48.27 ± 1.59^c^

TSS, total soluble solids; TA, titratable acidity; CAE, citric acid equivalent; GE, glucose equivalent. Data are presented as mean ± standard deviation of three independent measurements. Different lowercase letters within the same column indicate significant differences among treatments according to Tukey's HSD test (*p* < 0.05).

In contrast, both WB and RF treatments significantly increased TSS and TA compared with CK. This result is consistent with the findings of (Adulvitayakorn et al., 2020), who observed increased TSS and TA in sugarcane juice after different heat treatments. Among the RF-treated samples, RF-9 showed significantly higher TSS and TA values than RF-11 and RF-13. This may be related to the stronger heating intensity at the smaller electrode gap, which promoted greater disruption of apple tissue and facilitated the release of intracellular soluble components into the juice. Under RF treatment, faster heating and greater tissue disruption may have promoted the release of sugars and organic acids from the pulp into the juice, thereby increasing TSS and TA (Liu et al., 2024). Although these changes were accompanied by some decrease in pH, the pH values varied only slightly among treatments overall, indicating that treatment-related differences in acidity were more evident in TA than in pH. A similar trend was observed for total sugar content. The increase in total sugar content may be associated with the breakdown of cellular structures during both RF and WB treatments, which promoted the release of soluble sugars from the apple pulp into the juice. This trend was also consistent with the higher phenolic content observed in RF-treated samples, indicating that RF treatment promoted the release of multiple soluble components into the juice.

The organic acid profile in juice after thermal treatment of the solid-liquid mixture.

Organic acids are important contributors to the flavor of apple juice, especially citric acid and malic acid, which largely determine its sour taste. The overall quality of apple juice is closely related to both the content and composition of these acids (Ma et al., 2018; Wu et al., 2007). As shown in Table 2, malic acid was the dominant organic acid in apple juice, followed by citric acid, which agrees with previous reports by Wu et al. (2007). WB and RF treatments reduced oxalic acid and malic acid contents, which may be due to their sensitivity to heat. In contrast, RF treatment increased citric acid and succinic acid contents compared with CK and WB. One possible explanation is that RF treatment promoted tissue disruption and solute redistribution, which may have facilitated the release of intracellular organic acids into the juice matrix (Sun et al., 2025). In the RF-11 group, citric acid reached 241.92 μg/g and succinic acid reached 35.64 μg/g. These changes were generally consistent with the higher TA values observed after RF treatment. However, the pH values varied only within a relatively narrow range among treatments, indicating that treatment-related differences in acidity were reflected more clearly by TA and organic acid composition than by pH alone. In the present study, no direct measurements were performed on enzymes involved in organic acid metabolism or metabolic flux. Therefore, these results are interpreted mainly as possible consequences of tissue disruption and solute redistribution during processing. At the same time, we cannot exclude other possibilities. For example, the observed profile may be partly related to changes in citrate-associated metabolism under RF treatment, or it may simply reflect greater cellular disruption and release of intracellular acids after longer exposure. However, these possibilities were not directly examined in this study (Li et al., 2024). Prolonged RF treatment may also promote the degradation of some organic acids, as suggested by the reduced citric acid and succinic acid contents in the RF-13 group. In addition, both WB and RF-13 showed relatively higher quinic acid contents. Because quinic acid may contribute to bitterness, this change could negatively affect flavor quality (Frank et al., 2006). Overall, RF treatment was associated with changes in the organic acid profile of apple juice, including higher citric acid and succinic acid contents under some RF conditions, while shorter heating times may also have helped reduce the loss of certain heat-sensitive components.

**Table 2:** Organic acid contents (μg/g) in juice obtained under different treatments.

	Oxalic acid	Citric acid	Malic acid	Quinic acid	Tartaric acid	Succinic acid
CK	70.36 ± 5.31ᵃ	207.15 ± 9.33ᶜ	703.77 ± 38.55ᵃ	41.26 ± 2.98ᵃ	15.56 ± 1.25ᵃ	21.38 ± 2.03ᶜ
WB	47.12 ± 3.66ᵈ	192.55 ± 10.34ᶜ	512.65 ± 17.33ᶜ	37.23 ± 3.21ᵃ	-	29.56 ± 1.65ᵇ
RF-9	57.89 ± 2.01ᵇ	217.22 ± 13.21ᵇ	554.75 ± 25.41ᶜ	35.65 ± 3.84ᵃ	11.27 ± 0.94ᵇ	33.87 ± 2.44ᵃ
RF-11	53.45 ± 1.08ᶜ	241.92 ± 17.56ᵃ	597.66 ± 27.86ᵇ	33.58 ± 2.42ᵇ	–	35.64 ± 2.31ᵃ
RF-13	50.55 ± 2.04ᶜ	221.32 ± 9.62ᵇ	588.21 ± 23.99ᵇ	39.64 ± 1.68ᵃ	-	25.64 ± 0.87ᵇ

-, not detected. Data are presented as mean ± standard deviation of three independent measurements. Different lowercase letters within the same column indicate significant differences among treatments according to Tukey's HSD test (p < 0.05).

SEM of structural changes after thermal treatment of the solid-liquid mixture.

**Fig. 5:**
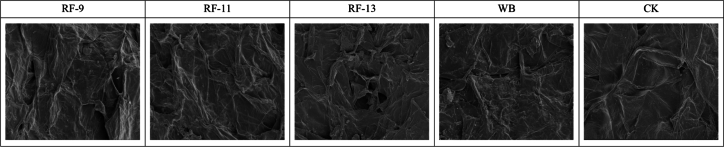
Scanning electron micrographs (2000× magnification) of apple pomace after different treatments: CK (control), RF-9, RF-11, RF-13, and WB.

SEM was used to observe the structural changes in the surface morphology of apple pomace after different treatments, providing insights into the physical breakdown of the tissue and its relationship with the release of bioactive compounds. As shown in Fig. 5, CK exhibited a smooth, compact surface with minimal disruption, which corresponds to its relatively lower levels of polyphenols, flavonoids, and antioxidant capacity. In contrast, the RF treatments, particularly RF-9, caused more pronounced changes, with the surface becoming rougher and exhibiting more cracks, wrinkles, and areas of local porosity. This suggests more extensive tissue breakdown in the RF-9 sample. This observation was consistent with the higher levels of TPC, TFC, TSS, and total sugar measured in the corresponding juice samples. The RF-11 and RF-13 treatments also resulted in structural changes, although to a lesser extent, with RF-13 showing a more intact surface compared to RF-9. The water bath (WB) treatment caused moderate surface relaxation and some wrinkling, but less pronounced damage compared to the RF treatments, which may explain its more limited increase in polyphenols and total sugars. These SEM results may help explain the compositional differences observed among treatments.

Volatile Compounds.

The *E*-nose aroma preservation in juice after thermal treatment of the solid-liquid mixture.

**Fig. 6:**
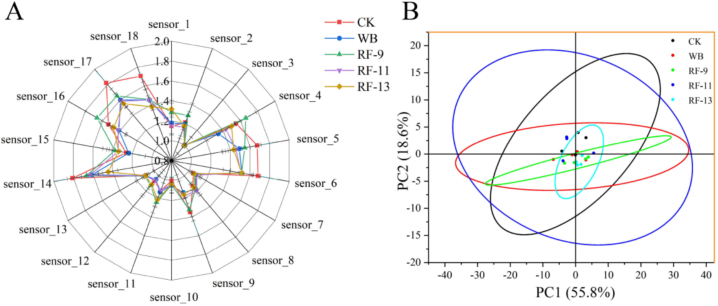
Electronic nose analysis of apple juice under different treatments. (A) Radar plot of normalized sensor response ratios at the stable detection stage. (B) PCA score plot of volatile profiles.

The electronic nose mimics the olfactory system of humans or animals and is used for the overall evaluation of food aromas (Haddi et al., 2014). To study the flavor characteristics of apple juice after different treatments, this research employed an electronic nose system equipped with 18 sensors to comprehensively assess the flavor. Table 3 lists the 18 types of metal oxide gas sensors and their corresponding response values. Based on Fig. 6A, the chart shows that the CK group had the highest response values for most sensors. This could be due to the fact that the CK samples were not subjected to heat treatment, thereby retaining most of the original aromas (Kato et al., 2003). The RF-9 group was closest to the CK group in terms of response values on most sensors. This could be attributed to the short RF heating time, which had a minimal impact on the aroma (Zhu et al., 2021). In particular, sensor_4 and sensor_16 in the RF-9 treatment group showed an increase in response values. The corresponding strong alternating electric field from the RF may have a specific effect on sulfur compounds (Nandakumar et al., 2018). Fig. 6B demonstrates a clear separation between the untreated control group (CK) and the water bath-treated group (WB) along the PC1 axis, indicating substantial differences between these two processing methods. This indicates that the aroma profile of the WB sample differed from that of CK. By comparison, the RF-treated groups were located closer to CK in the PCA plot.

**Table 3:** Sensor array and sensor type of the electronic nose.

Sensor	Sensor type
Sensor_1	Short-chain alkanes
Sensor_2	Carbonaceous matter
Sensor_3	Hydrogen
Sensor_4	Potassium sulfide
Sensor_5	Nitrogenous substances
Sensor_6	Aldoketones
Sensor_7	Short-chain paraffin flammable gas
Sensor_8	Liquefied gas
Sensor_9	Alkanes, alcohols, ketones
Sensor_10	Hydrogens, hydrogen
Sensor_11	Alkanes, carbon monoxide
Sensor_12	Partial organic solvent
Sensor_13	Short-chain alkanes
Sensor_14	Short-chain alkanes
Sensor_15	Carbon-based substances, alcohols, aldehydes
Sensor_16	Sulfur compound
Sensor_17	Nitrogenous substances
Sensor_18	Aldehydes and ketones organic

These results suggest that short, high-intensity RF had only a limited impact on the overall flavor of the juice. To explore whether short, high-intensity RF treatment induces subtle changes in key volatile organic compounds (VOCs) responsible for flavor—such as esters, aldehydes, terpenes, or phenolic derivatives—we conducted a comparative GC–MS profiling between treated and untreated juice samples.

The volatile compounds in apple juice (GC–MS).

The identification of volatile components and the comparison of their relative concentrations are crucial for evaluating the impact of different processing methods on the aroma of apple juice. Together with electronic nose measurements, this establishes the aroma profile of the juice. Aroma intensity is a key quality indicator and an important factor in consumer attraction (Guo et al., 2020). As shown in Table 4, a total of 23 volatile compounds were detected in the apple juice samples, including 5 alcohols, 8 esters, 2 phenols, 4 aldehydes, 3 ketones, and 1 other compound. In previous studies on apple juice, 19 of these compounds were identified as volatiles (Guo et al., 2020; Wu et al., 2017). Esters and alcohols accounted for the highest proportion, consistent with the typical aroma profile of apple juice (Coelho et al., 2021). Among the heated samples, RF-9 generally retained more characteristic volatile compounds than WB, RF-11, and RF-13, suggesting a milder impact on the aroma profile under this condition.

**Table 4:** Volatile compounds identified in apple juice under different treatments.

Compounds	Rt	Concentration (μg/L)					RI	Identification
Alcohols		CK	WB	RF-9	RF-11	RF-13		
1-Butanol	2.271	18.36 ± 0.20b	15.96 ± 0.14c	16.33 ± 0.38b	15.84 ± 0.58c	15.03 ± 0.35d	655	MS, RI
2-Methyl-1-butanol	3.306	36.84 ± 0.91a	23.51 ± 3.73d	30.82 ± 0.11b	27.20 ± 1.66c	20.17 ± 2.16e	718	MS, RI
(Z)-2-Hexen-1-ol	7.494	3.50 ± 0.16c	3.34 ± 0.20c	4.57 ± 0.32b	5.21 ± 0.15a	3.41 ± 0.26c	864	MS, RI
1-Hexanol	7.596	36.84 ± 0.91a	35.51 ± 3.73b	35.82 ± 0.11b	27.20 ± 1.66d	34.17 ± 2.16c	866	MS, RI
3-Methyl-1-hexanol	8.048	5.88 ± 0.34c	5.42 ± 0.31d	6.82 ± 0.58b	7.50 ± 0.25a	5.09 ± 0.34e	879	MS, RI
Esters								
Butyl acetate	5.17	102.81 ± 3.71a	44.01 ± 0.35b	48.88 ± 3.66b	47.06 ± 3.89b	42.06 ± 3.89b	804	MS, RI
2-Methylbutyl acetate	6.71	60.04 ± 3.13a	30.59 ± 2.38c	37.75 ± 0.75b	36.02 ± 1.08b	31.90 ± 2.62c	844	MS, RI
Hexyl acetate	11.43	228.65 ± 9.13a	150.05 ± 3.73c	179.24 ± 10.01b	161.97 ± 4.45bc	151.71 ± 20.16c	960	MS, RI
Isobutyl butyrate	14.946	5.66 ± 0.53a	–	5.62 ± 0.28a	4.97 ± 0.3b	–	1045	MS, RI
Butyl hexanoate	16.12	17.32 ± 1.13a	9.05 ± 0.64c	13.90 ± 1.84b	10.46 ± 0.09c	9.64 ± 0.07c	1073	MS, RI
Hexyl 2-methylbutyrate	22.413	2.537 ± 1.78a	2.229 ± 0.62b	–	–	1.662 ± 1.61c	1239	MS, RI
2,2,4-Trimethyl-1,3-pentanediol diisobutyrate	26.725	11.88 ± 1.05a	1.64 ± 0.16c	0.46 ± 0.03d	0.73 ± 0.05d	5.89 ± 0.37b	1377	MS, RI
Hexyl hexanoate	27.073	1.46 ± 0.12a	0.89 ± 0.07c	1.07 ± 0.08b	0.64 ± 0.03d	0.92 ± 0.09c	1389	MS, RI
Phenols								
4-(1,1-Dimethylpropyl)phenol	27.262	6.69 ± 0.25a	5.29 ± 0.52b	3.71 ± 0.09c	4.31 ± 0.39c	6.46 ± 0.31a	1396	MS, RI
2,4-Di-tert-butylphenol	29.495	4.05 ± 0.32a	3.27 ± 0.08b	2.33 ± 0.19d	2.72 ± 0.22c	2.70 ± 0.25c	1514	MS, RI
Aldehydes								
Hexanal	4.89	47.16 ± 6.55a	38.18 ± 3.61c	31.19 ± 2.46d	42.27 ± 1.06b	37.90 ± 4.99c	794	MS, RI
(E)-2-Hexenal	6.88	102.81 ± 3.71a	48.88 ± 3.66b	44.01 ± 0.35b	32.96 ± 3.09c	47.06 ± 3.89b	848	MS, RI
Nonanal	17.4	6.12 ± 0.12a	4.88 ± 0.39b	6.18 ± 0.57a	6.03 ± 0.53a	5.01 ± 0.42b	1105	MS, RI
Decanal	21.251	8.44 ± 0.03a	8.46 ± 0.07a	5.95 ± 0.09b	5.58 ± 0.02c	8.83 ± 0.26a	1206	MS, RI
Ketones								
6-Methyl-5-hepten-2-one	12.625	6.60 ± 0.61a	3.18 ± 0.10d	3.96 ± 0.18c	3.60 ± 0.08c	4.79 ± 0.22b	988	MS, RI
(E)-6,10-Dimethyl-5,9-undecadien-2-one	28.451	1.75 ± 0.03a	1.10 ± 0.04c	1.24 ± 0.1b	0.86 ± 0.08d	1.16 ± 0.10b	1329	MS, RI
β-Damascenone	24.268	14.57 ± 1.23a	8.85 ± 0.37c	10.64 ± 0.18b	6.41 ± 0.07d	9.19 ± 0.59c	1235	MS, RI
Others								
α-Farnesene	29.436	4.14 ± 0.31a	2.76 ± 0.26c	3.36 ± 0.20b	3.60 ± 0.23b	3.13 ± 0.13c	1635	MS, RI

Rt, retention time; RI, retention index; MS, mass spectrometry. -, not detected. Data are presented as mean ± standard deviation of three independent measurements. Different lowercase letters within the same row indicate significant differences among treatments according to Tukey's HSD test (p < 0.05).

Esters are key compounds responsible for the sweet and fruity aromas of apple juice. Among them, butyl acetate, hexyl acetate, butyric acid hexyl ester, hexyl butyrate, and 2-methylbutyl acetate are considered characteristic aroma compounds of apple juice (Guo et al., 2020). The total ester content was highest in the CK group (e.g., Hexyl acetate at 228.65 μg/L), and significantly reduced in the RF-9 and WB groups (decreasing by 33.33%–44.58%), indicating that heat treatment leads to ester degradation. Among the treatments, RF-13 and WB caused the greatest loss of esters, likely due to prolonged heating. The RF-9 group retained significantly more esters than the other heated treatments, especially for several characteristic fruity esters, which may be related to the shorter heating time at the 9 cm electrode gap. This result was also consistent with the electronic nose analysis, where RF-9 was located closer to CK than the other treated groups. However, the results for 2,2,4-Trimethyl-1,3-pentanediol diisobutyrate showed the opposite trend, which may be due to the destruction and volatilization of long-chain esters requiring more powerful instantaneous energy.

Alcohols are degradation products of unsaturated fatty acids and typically have higher odor thresholds. They are also considered the second largest contributors to apple aroma, imparting a fresh and sweet flavor to apple juice (Bult et al., 2002). 1-Hexanol, 1-butanol, and 2-methyl-1-butanol are considered characteristic aroma compounds of apple juice. Heat treatment primarily leads to the loss of alcohols, with more significant losses occurring as the heating time increases. RF-9 retained higher levels of several alcohol compounds than RF-11, RF-13, and WB, which may be related to its shorter heating time. Some alcohols, such as (Z)-2-Hexen-1-ol and 3-Methyl-1-hexanol, showed an increasing trend, which may be related to the cell disruption induced by RF. Overall, RF-9 showed a better retention of several alcohol compounds than WB, RF-11, and RF-13, indicating that the shorter RF treatment was more favorable for preserving these fresh and sweet aroma notes.

The fresh grassy aroma of apple juice is primarily contributed by aldehydes, which are produced during enzymatic browning after the apples are crushed (Guo et al., 2020). Hexanal and (E)-2-hexenal are the aldehydes present in relatively high concentrations in apple juice. Under high-intensity radio-frequency conditions, both aldehydes demonstrated a more significant decrease compared to other treatment conditions. This suggests that aldehydes were also affected by the processing conditions, although the extent of change differed among treatments. Compared with the other heated groups, RF-9 showed a more pronounced reduction in several aldehydes, indicating that RF blanching altered not only the retention of fruity volatiles, but also the balance of green-note compounds in the juice aroma profile (Wang et al., 2022).

Taken together, these results suggest that although thermal treatment changed the volatile composition of apple juice, RF-9 retained an aroma profile closer to CK than the other heated treatments.

The sensory evaluation of apple juice.

**Fig. 7:**
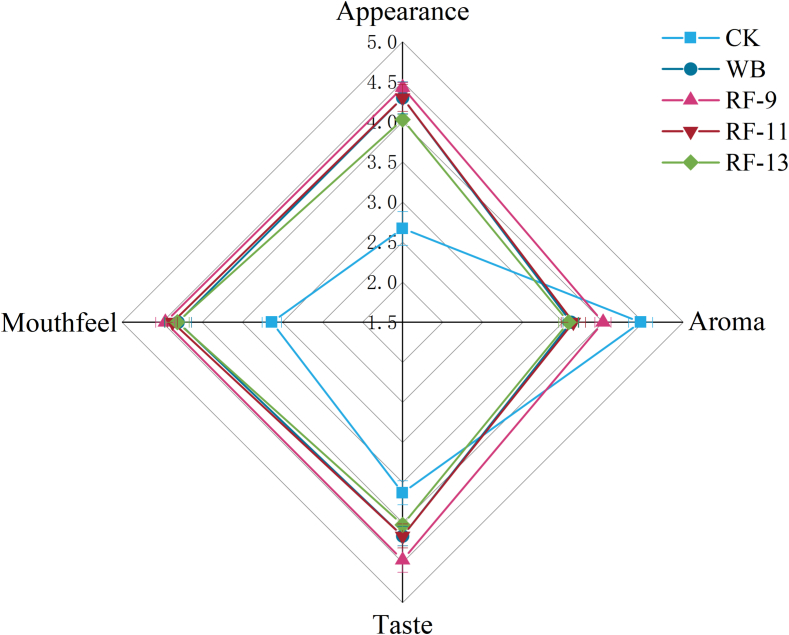
Sensory evaluation of apple juice under different treatments.

The sensory properties of apple juice were evaluated from four aspects (Table 5), and the results are shown in Fig. 7. Among the treated samples, RF-9 showed the highest overall sensory score. In terms of appearance, all treated juices received relatively good scores, which was in agreement with the color results. Aroma scores decreased after thermal treatment, but RF-9 (4.00 ± 0.10) remained closer to CK (4.47 ± 0.15) than the other treated samples. This suggests that RF-9 better retained the original aroma characteristics of apple juice. Compared with CK, the heat-treated samples showed higher taste scores, which may be related to changes in acid composition after treatment. Mouthfeel scores were also higher in the treated groups, although no significant differences were found among the four heat-treated samples. This may be associated with changes in juice texture after processing. Since the sensory evaluation in this study was used as a preliminary screening, these results should be interpreted with caution. Overall, the sensory observations were generally consistent with the physicochemical and volatile analyses, and RF-9 showed the most favorable sensory performance among the treated samples.

**Table 5:** Sensory attributes and definitions of apple juice.

Sensory descriptor	Definition
Appearance	Uniform pale yellow-amber, free from impurities
Aroma	The Natural Fragrance of Apples
Taste	Balanced sweet-sour profile with natural apple flavor
Mouthfeel	A refreshing mouthfeel, free from grittiness or granular texture

## 4 Conclusion

This study evaluated the effects of radio frequency (RF) blanching on a comminuted apple solid-liquid mixture, with particular attention to the release of bioactive phytochemicals and the antioxidant capacity of the resulting pressed juice, in comparison with conventional water-bath (WB) treatment. The results showed that rapid RF heating improved the inactivation of polyphenol oxidase and peroxidase, thereby reducing enzymatic browning and increasing juice brightness. RF treatment also promoted the transfer of soluble compounds from the disrupted solid phase into the liquid phase, resulting in higher contents of phenolics and flavonoids in the pressed juice and, consequently, greater antioxidant capacity. Among the tested RF conditions, RF-9 showed the shortest heating time and was associated with higher phenolic and flavonoid contents, higher antioxidant capacity, and better retention of several volatile compounds than the other heated treatments. Scanning electron microscopy further showed that RF-treated pomace exhibited more severe tissue disruption, which may have increased mass transfer and facilitated the release of intracellular phytochemicals. In addition, RF treatment retained higher levels of key fruity esters and alcohols, while reducing several aldehydes associated with flavor deterioration, resulting in an aroma profile closer to that of fresh juice. Changes in organic acids were also observed, which may be related to cell disruption and solute redistribution during treatment. Preliminary sensory evaluation was generally consistent with these results, showing that juice treated under the RF-9 condition was closer to the untreated sample in aroma and mouthfeel than the other heated samples.

Overall, RF blanching appears to be a promising pretreatment for improving the nutritional and sensory quality of apple juice produced from comminuted solid-liquid mixtures. Although this study clearly demonstrated the effects of RF blanching on polyphenol release and juice quality, the underlying mechanisms were not directly elucidated. Further studies are needed to clarify energy input and process regulation during RF treatment, and to evaluate scale-up feasibility and quality stability under industrial conditions.

## CRediT authorship contribution statement

**Yidian Li:** Writing – review & editing, Writing – original draft, Methodology, Data curation. **Shikang Tang:** Visualization, Methodology, Investigation. **Wenjie Ding:** Data curation, Conceptualization. **Bimal Chitrakar:** Visualization. **Baoguo Xu:** Visualization. **Junjie Yi:** Writing – review & editing, Validation, Supervision, Resources, Project administration. **Xuejiao Wang:** Visualization. **Chaofan Guo:** Writing – review & editing, Supervision, Resources, Project administration, Funding acquisition.

## Ethical statement

This study involved sensory evaluation conducted by a trained panel. All procedures involving human participants were performed in accordance with the ethical guidelines of Kunming University of Science and Technology and the principles of the Declaration of Helsinki. For this type of low-risk sensory study, separate ethical review and formal documentation for the sensory evaluation itself were not required under institutional regulations. Nevertheless, the main project associated with this work, supported by the National Natural Science Foundation of China (Grant No. 32502269), had previously been approved by the Medical Ethics Committee of Kunming University of Science and Technology (Approval No. KMUST-MEC-2026-080). Informed consent was obtained from all participants prior to the sensory evaluation. Participants were informed of the study procedures and their right to decline participation or withdraw at any time without penalty. All personal information was kept confidential, and participant anonymity was maintained throughout the study.

## Funding

National Natural Science Foundation of China (grant NO. 32502269).

Young talents from Yunnan Province's “Prosperous Yunnan Talent Support Plan”(Project No. XDYC-QNRC-2022-0569).

## Declaration of competing interest

The authors declare that they have no known competing financial interests or personal relationships that could have appeared to influence the work reported in this paper.

## Data Availability

Data will be made available on request.
